# Bis(1*H*-benzotriazole-4-sulfonato-κ^2^ *N* ^3^,*O*)(2,2′-bipyridyl-κ^2^ *N*,*N*′)cadmium

**DOI:** 10.1107/S160053681104596X

**Published:** 2011-11-05

**Authors:** Xiao-Hong Zhu, Xiao-Chun Cheng

**Affiliations:** a Faculty of Life Science and Chemical Engineering, Huaiyin Institute of Technology, Huaian 223003, People’s Republic of China

## Abstract

In the title complex, [Cd(C_6_H_4_N_3_O_3_S)_2_(C_10_H_8_N_2_)], the Cd^2+^ cation is located on a twofold rotation axis and is coordinated by two N and two O atoms from two symmetry-related benzotriazole-4-sulfonate anions and two N atoms from a 2,2-bipyridyl ligand, displaying a distorted CdN_4_O_2_ octa­hedral geometry. The crystal structure is stabilized by N—H⋯O and C—H⋯O hydrogen-bonding inter­actions.

## Related literature

For a related structure, see: Xia *et al.* (2010 ▶).
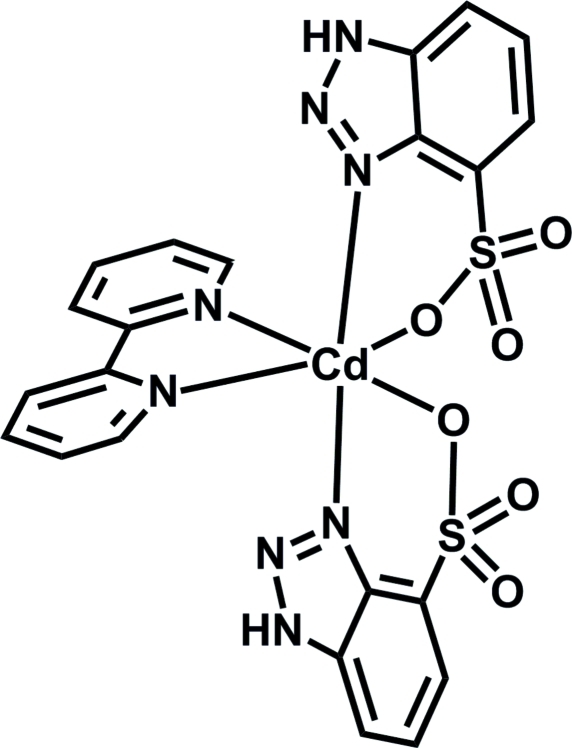


## Experimental

### 

#### Crystal data

[Cd(C_6_H_4_N_3_O_3_S)_2_(C_10_H_8_N_2_)]
*M* *_r_* = 664.95
Monoclinic, 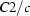

*a* = 8.148 (4) Å
*b* = 17.207 (7) Å
*c* = 17.720 (8) Å
β = 103.29 (1)°
*V* = 2417.8 (19) Å^3^
*Z* = 4
Mo *K*α radiation
μ = 1.14 mm^−1^
*T* = 293 K
0.20 × 0.20 × 0.20 mm

#### Data collection

Bruker SMART APEXII CCD diffractometer
Absorption correction: multi-scan (*SADABS*; Sheldrick, 1996 ▶) *T*_min_ = 0.805, *T*_max_ = 0.805
8519 measured reflections
3009 independent reflections
2866 reflections with *I* > 2σ(*I*)
*R*_int_ = 0.073

#### Refinement

*R*[*F* ^2^ > 2σ(*F* ^2^)] = 0.028
*wR*(*F* ^2^) = 0.075
*S* = 1.05
3009 reflections
177 parameters
H-atom parameters constrained
Δρ_max_ = 0.82 e Å^−3^
Δρ_min_ = −0.64 e Å^−3^

### 

Data collection: *APEX2* (Bruker, 2008 ▶); cell refinement: *SAINT* (Bruker, 2008 ▶); data reduction: *SAINT*; program(s) used to solve structure: *SHELXS97* (Sheldrick, 2008 ▶); program(s) used to refine structure: *SHELXL97* (Sheldrick, 2008 ▶); molecular graphics: *DIAMOND* (Brandenburg, 2000 ▶); software used to prepare material for publication: *SHELXTL* (Sheldrick, 2008 ▶).

## Supplementary Material

Crystal structure: contains datablock(s) I, global. DOI: 10.1107/S160053681104596X/pv2474sup1.cif

Structure factors: contains datablock(s) I. DOI: 10.1107/S160053681104596X/pv2474Isup2.hkl

Supplementary material file. DOI: 10.1107/S160053681104596X/pv2474Isup3.cdx

Additional supplementary materials:  crystallographic information; 3D view; checkCIF report

## Figures and Tables

**Table 1:** Hydrogen-bond geometry (Å, °)

*D*—H⋯*A*	*D*—H	H⋯*A*	*D*⋯*A*	*D*—H⋯*A*
N3—H3*N*⋯O2^i^	0.93	1.86	2.776 (3)	167
C3—H3⋯O3^ii^	0.93	2.55	3.255 (3)	133
C7—H7⋯O2^iii^	0.93	2.56	3.204 (3)	127

Symmetry codes: (i) 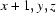; (ii) 
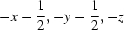
; (iii) 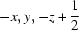.

**Fig. 1.**
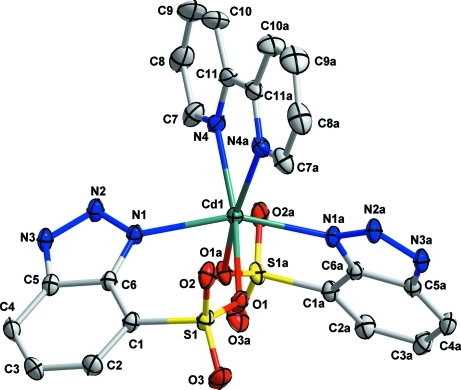
The coordination environment of Cd ion in the title complex with the ellipsoids drawn at the 30% probability level. The hydrogen atoms have been omitted for clarity. symmetry code: a: -x, y, -z+1/2.

## supplementary crystallographic information

### Comment

Benzotriazole-4-sulfonic acid is often used as a ligand to synthesize complexes for its variable coordination modes. Herein, we report the crystal structure of title complex. The asymmetric unit consists of half of a cadmium ion, half of a 2,2-bipyridyl molecule, and a benzotriazole-4-sulfonate anion. The Cd ion is located on a two fold axis and coordinated by two N atoms from two different benzotriazole-4-sulfonate anions, two N atoms from 2,2-bipyridyl molecule, and two sulfonate O atoms from two different benzotriazole-4-sulfonate anions, displaying a distorted CdN_4_O_2_ octahedral geometry (Fig. 1). Both benzotriazole-4-sulfonate and 2,2-bipyridyl display bidentate chelating coordinating mode. In the crystal structure, there exist O—H···N and C—H···O hydrogen bonds (Table 1). Sulfonate O atoms as hydrogen bonding acceptor play a very important role in the formation of these hydrogen bonding interactions.

### Experimental

A mixture of cadmium perchlorate hexahydrate (83.9 mg, 0.2 mmol), benzotriazole-4-sulfonic acid (39.8 mg, 0.2 mmol), 2,2-bipyridyl (31.2 mg, 0.2 mmol) and potassium hydroxide (11.2 mg, 0.2 mmol) in 12 ml H_2_O was sealed in a 16 ml Teflon-lined stainless steel container and heated to 393 K for 3 days. After cooling to room temperature, colorless block crystals of the title complex were obtained.

### Refinement

The hydrogen atoms bonded to C atoms were located in geometrically idealized positions and constrained to ride on their parent atoms, with C—H = 0.93 Å and *U*_iso_(H) = 1.2*U*_eq_(C). The hydrogen atom bonded to N3 was found from a difference Fourier map and fixed at that positio with *U*_iso_(H) = 1.2*U*_eq_(N).

### Crystal data

**Table:**

[Cd(C_6_H_4_N_3_O_3_S)_2_(C_10_H_8_N_2_)]	*F*(000) = 1328
*M**_r_* = 664.95	*D*_x_ = 1.827 Mg m^−^^3^
Monoclinic, *C*2/*c*	Mo *K*α radiation, λ = 0.71073 Å
Hall symbol: -C 2yc	Cell parameters from 6866 reflections
*a* = 8.148 (4) Å	θ = 2.4–28.4°
*b* = 17.207 (7) Å	µ = 1.14 mm^−^^1^
*c* = 17.720 (8) Å	*T* = 293 K
β = 103.29 (1)°	Block, colorless
*V* = 2417.8 (19) Å^3^	0.20 × 0.20 × 0.20 mm
*Z* = 4	

### Data collection

**Table:**

Bruker SMART APEXII CCD diffractometer	3009 independent reflections
Radiation source: fine-focus sealed tube	2866 reflections with *I* > 2σ(*I*)
graphite	*R*_int_ = 0.073
phi and ω scans	θ_max_ = 28.4°, θ_min_ = 2.4°
Absorption correction: multi-scan (*SADABS*; Sheldrick, 1996)	*h* = −9→10
*T*_min_ = 0.805, *T*_max_ = 0.805	*k* = −22→22
8519 measured reflections	*l* = −23→17

### Refinement

**Table:**

Refinement on *F*^2^	Primary atom site location: structure-invariant direct methods
Least-squares matrix: full	Secondary atom site location: difference Fourier map
*R*[*F*^2^ > 2σ(*F*^2^)] = 0.028	Hydrogen site location: inferred from neighbouring sites
*wR*(*F*^2^) = 0.075	H-atom parameters constrained
*S* = 1.05	*w* = 1/[σ^2^(*F*_o_^2^) + (0.0361*P*)^2^ + 1.8012*P*] where *P* = (*F*_o_^2^ + 2*F*_c_^2^)/3
3009 reflections	(Δ/σ)_max_ < 0.001
177 parameters	Δρ_max_ = 0.82 e Å^−^^3^
0 restraints	Δρ_min_ = −0.64 e Å^−^^3^

### Special details

**Table:**

Geometry. All e.s.d.'s (except the e.s.d. in the dihedral angle between two l.s. planes) are estimated using the full covariance matrix. The cell e.s.d.'s are taken into account individually in the estimation of e.s.d.'s in distances, angles and torsion angles; correlations between e.s.d.'s in cell parameters are only used when they are defined by crystal symmetry. An approximate (isotropic) treatment of cell e.s.d.'s is used for estimating e.s.d.'s involving l.s. planes.
Refinement. Refinement of *F*^2^ against ALL reflections. The weighted *R*-factor *wR* and goodness of fit *S* are based on *F*^2^, conventional *R*-factors *R* are based on *F*, with *F* set to zero for negative *F*^2^. The threshold expression of *F*^2^ > σ(*F*^2^) is used only for calculating *R*-factors(gt) *etc*. and is not relevant to the choice of reflections for refinement. *R*-factors based on *F*^2^ are statistically about twice as large as those based on *F*, and *R*- factors based on ALL data will be even larger.

### Fractional atomic coordinates and isotropic or equivalent isotropic displacement parameters (Å^2^)

**Table:**

	*x*	*y*	*z*	*U*_iso_*/*U*_eq_	
C1	−0.0973 (2)	−0.10519 (11)	0.06169 (11)	0.0286 (3)	
C2	−0.1066 (3)	−0.15816 (14)	0.00234 (12)	0.0388 (4)	
H2	−0.2059	−0.1860	−0.0150	0.047*	
C3	0.0298 (3)	−0.17160 (14)	−0.03310 (13)	0.0449 (5)	
H3	0.0179	−0.2085	−0.0724	0.054*	
C4	0.1784 (3)	−0.13203 (14)	−0.01131 (12)	0.0400 (5)	
H4	0.2683	−0.1409	−0.0344	0.048*	
C5	0.1872 (2)	−0.07701 (12)	0.04815 (11)	0.0297 (4)	
C6	0.0555 (2)	−0.06363 (10)	0.08486 (10)	0.0252 (3)	
C7	0.3344 (3)	0.12261 (16)	0.31089 (13)	0.0445 (5)	
H7	0.3858	0.0742	0.3206	0.053*	
C8	0.4326 (4)	0.18893 (19)	0.32763 (15)	0.0573 (7)	
H8	0.5472	0.1855	0.3504	0.069*	
C9	0.3560 (4)	0.26011 (18)	0.30959 (19)	0.0681 (9)	
H9	0.4197	0.3055	0.3180	0.082*	
C10	0.1860 (4)	0.26376 (15)	0.27930 (18)	0.0601 (7)	
H10	0.1331	0.3116	0.2676	0.072*	
C11	0.0927 (3)	0.19536 (12)	0.26615 (11)	0.0376 (4)	
Cd1	0.0000	0.017130 (9)	0.2500	0.02575 (8)	
N1	0.1089 (2)	−0.00941 (9)	0.14217 (10)	0.0281 (3)	
N2	0.2645 (2)	0.01101 (11)	0.14125 (11)	0.0340 (4)	
N3	0.3117 (2)	−0.02916 (11)	0.08527 (10)	0.0329 (3)	
H3N	0.4172	−0.0247	0.0738	0.039*	
N4	0.1685 (2)	0.12587 (10)	0.28136 (10)	0.0338 (3)	
O1	−0.18547 (17)	−0.07919 (9)	0.19035 (8)	0.0335 (3)	
O2	−0.3547 (2)	−0.02209 (10)	0.07349 (10)	0.0410 (4)	
O3	−0.3666 (2)	−0.16108 (11)	0.09518 (10)	0.0492 (4)	
S1	−0.26613 (5)	−0.09167 (3)	0.10818 (3)	0.02965 (11)	

### Atomic displacement parameters (Å^2^)

**Table:**

	*U*^11^	*U*^22^	*U*^33^	*U*^12^	*U*^13^	*U*^23^
C1	0.0243 (8)	0.0342 (9)	0.0272 (8)	−0.0016 (7)	0.0055 (6)	0.0006 (7)
C2	0.0396 (11)	0.0449 (11)	0.0311 (9)	−0.0085 (9)	0.0066 (8)	−0.0059 (8)
C3	0.0592 (14)	0.0445 (12)	0.0351 (10)	−0.0033 (10)	0.0190 (10)	−0.0119 (9)
C4	0.0450 (11)	0.0463 (11)	0.0340 (10)	0.0051 (9)	0.0200 (9)	−0.0028 (9)
C5	0.0265 (8)	0.0363 (9)	0.0277 (8)	0.0035 (7)	0.0090 (7)	0.0043 (7)
C6	0.0231 (8)	0.0290 (8)	0.0239 (7)	0.0021 (6)	0.0062 (6)	0.0020 (6)
C7	0.0413 (11)	0.0528 (13)	0.0381 (10)	−0.0126 (10)	0.0065 (9)	0.0050 (9)
C8	0.0529 (15)	0.0773 (19)	0.0404 (12)	−0.0292 (14)	0.0078 (11)	−0.0097 (12)
C9	0.082 (2)	0.0547 (17)	0.0692 (18)	−0.0351 (15)	0.0195 (16)	−0.0240 (14)
C10	0.082 (2)	0.0328 (11)	0.0678 (17)	−0.0149 (12)	0.0225 (15)	−0.0123 (11)
C11	0.0582 (13)	0.0297 (9)	0.0283 (9)	−0.0072 (9)	0.0169 (8)	−0.0039 (7)
Cd1	0.02654 (11)	0.02444 (11)	0.02832 (11)	0.000	0.01050 (7)	0.000
N1	0.0234 (7)	0.0339 (8)	0.0281 (7)	−0.0031 (6)	0.0082 (6)	−0.0019 (6)
N2	0.0260 (8)	0.0460 (10)	0.0319 (8)	−0.0047 (6)	0.0104 (6)	−0.0015 (7)
N3	0.0233 (7)	0.0456 (9)	0.0318 (8)	−0.0004 (6)	0.0108 (6)	0.0007 (7)
N4	0.0384 (8)	0.0337 (8)	0.0301 (7)	−0.0077 (7)	0.0094 (6)	0.0010 (6)
O1	0.0291 (7)	0.0433 (8)	0.0283 (6)	−0.0103 (6)	0.0074 (5)	−0.0003 (5)
O2	0.0244 (7)	0.0578 (11)	0.0427 (8)	0.0073 (6)	0.0117 (6)	0.0099 (7)
O3	0.0450 (9)	0.0546 (10)	0.0499 (9)	−0.0275 (8)	0.0149 (7)	−0.0115 (8)
S1	0.0219 (2)	0.0385 (2)	0.0286 (2)	−0.00741 (16)	0.00572 (16)	−0.00111 (17)

### Geometric parameters (Å, °)

**Table:**

C1—C2	1.381 (3)	C9—H9	0.9300
C1—C6	1.412 (2)	C10—C11	1.391 (3)
C1—S1	1.774 (2)	C10—H10	0.9300
C2—C3	1.415 (3)	C11—N4	1.344 (3)
C2—H2	0.9300	C11—C11^i^	1.487 (5)
C3—C4	1.365 (3)	Cd1—N4	2.3114 (18)
C3—H3	0.9300	Cd1—N4^i^	2.3114 (18)
C4—C5	1.406 (3)	Cd1—O1	2.3267 (15)
C4—H4	0.9300	Cd1—O1^i^	2.3267 (15)
C5—N3	1.354 (3)	Cd1—N1	2.3293 (19)
C5—C6	1.397 (3)	Cd1—N1^i^	2.3293 (19)
C6—N1	1.374 (2)	N1—N2	1.319 (2)
C7—N4	1.334 (3)	N2—N3	1.336 (3)
C7—C8	1.386 (3)	N3—H3N	0.9310
C7—H7	0.9300	O1—S1	1.4690 (15)
C8—C9	1.378 (5)	O2—S1	1.4587 (17)
C8—H8	0.9300	O3—S1	1.4363 (16)
C9—C10	1.367 (5)		
			
C2—C1—C6	116.48 (18)	N4—Cd1—N4^i^	71.91 (10)
C2—C1—S1	121.87 (15)	N4—Cd1—O1	166.80 (5)
C6—C1—S1	121.63 (14)	N4^i^—Cd1—O1	100.35 (7)
C1—C2—C3	122.3 (2)	N4—Cd1—O1^i^	100.35 (7)
C1—C2—H2	118.8	N4^i^—Cd1—O1^i^	166.80 (5)
C3—C2—H2	118.8	O1—Cd1—O1^i^	89.15 (8)
C4—C3—C2	121.9 (2)	N4—Cd1—N1	92.23 (6)
C4—C3—H3	119.0	N4^i^—Cd1—N1	106.18 (6)
C2—C3—H3	119.0	O1—Cd1—N1	79.50 (6)
C3—C4—C5	115.9 (2)	O1^i^—Cd1—N1	84.43 (6)
C3—C4—H4	122.0	N4—Cd1—N1^i^	106.18 (6)
C5—C4—H4	122.0	N4^i^—Cd1—N1^i^	92.23 (6)
N3—C5—C6	104.12 (17)	O1—Cd1—N1^i^	84.43 (6)
N3—C5—C4	132.63 (19)	O1^i^—Cd1—N1^i^	79.50 (6)
C6—C5—C4	123.20 (18)	N1—Cd1—N1^i^	157.38 (8)
N1—C6—C5	108.02 (16)	N2—N1—C6	108.28 (16)
N1—C6—C1	131.83 (17)	N2—N1—Cd1	120.41 (13)
C5—C6—C1	120.12 (17)	C6—N1—Cd1	128.60 (12)
N4—C7—C8	122.2 (3)	N1—N2—N3	108.25 (16)
N4—C7—H7	118.9	N2—N3—C5	111.32 (17)
C8—C7—H7	118.9	N2—N3—H3N	123.9
C9—C8—C7	118.3 (3)	C5—N3—H3N	124.8
C9—C8—H8	120.9	C7—N4—C11	119.6 (2)
C7—C8—H8	120.9	C7—N4—Cd1	123.54 (16)
C10—C9—C8	119.7 (2)	C11—N4—Cd1	116.87 (15)
C10—C9—H9	120.1	S1—O1—Cd1	130.56 (8)
C8—C9—H9	120.1	O3—S1—O2	113.99 (11)
C9—C10—C11	119.5 (3)	O3—S1—O1	112.90 (10)
C9—C10—H10	120.3	O2—S1—O1	111.29 (10)
C11—C10—H10	120.3	O3—S1—C1	106.89 (10)
N4—C11—C10	120.7 (2)	O2—S1—C1	105.86 (9)
N4—C11—C11^i^	117.13 (12)	O1—S1—C1	105.16 (9)
C10—C11—C11^i^	122.13 (17)		
			
C6—C1—C2—C3	1.0 (3)	Cd1—N1—N2—N3	162.45 (12)
S1—C1—C2—C3	−177.47 (18)	N1—N2—N3—C5	0.0 (2)
C1—C2—C3—C4	−0.9 (4)	C6—C5—N3—N2	0.4 (2)
C2—C3—C4—C5	−0.3 (3)	C4—C5—N3—N2	−177.0 (2)
C3—C4—C5—N3	178.3 (2)	C8—C7—N4—C11	0.8 (3)
C3—C4—C5—C6	1.4 (3)	C8—C7—N4—Cd1	178.65 (18)
N3—C5—C6—N1	−0.6 (2)	C10—C11—N4—C7	1.4 (3)
C4—C5—C6—N1	177.08 (18)	C11^i^—C11—N4—C7	−179.5 (2)
N3—C5—C6—C1	−179.00 (16)	C10—C11—N4—Cd1	−176.55 (19)
C4—C5—C6—C1	−1.3 (3)	C11^i^—C11—N4—Cd1	2.5 (3)
C2—C1—C6—N1	−177.84 (19)	N4^i^—Cd1—N4—C7	−178.8 (2)
S1—C1—C6—N1	0.6 (3)	O1—Cd1—N4—C7	−123.2 (3)
C2—C1—C6—C5	0.1 (3)	O1^i^—Cd1—N4—C7	12.25 (18)
S1—C1—C6—C5	178.54 (14)	N1—Cd1—N4—C7	−72.49 (17)
N4—C7—C8—C9	−3.0 (4)	N1^i^—Cd1—N4—C7	94.20 (17)
C7—C8—C9—C10	2.9 (5)	N4^i^—Cd1—N4—C11	−0.92 (11)
C8—C9—C10—C11	−0.8 (5)	O1—Cd1—N4—C11	54.7 (3)
C9—C10—C11—N4	−1.4 (4)	O1^i^—Cd1—N4—C11	−169.89 (14)
C9—C10—C11—C11^i^	179.6 (3)	N1—Cd1—N4—C11	105.37 (15)
C5—C6—N1—N2	0.7 (2)	N1^i^—Cd1—N4—C11	−87.93 (15)
C1—C6—N1—N2	178.77 (19)	N4—Cd1—O1—S1	9.9 (3)
C5—C6—N1—Cd1	−160.36 (13)	N4^i^—Cd1—O1—S1	62.73 (12)
C1—C6—N1—Cd1	17.7 (3)	O1^i^—Cd1—O1—S1	−126.49 (14)
N4—Cd1—N1—N2	29.31 (15)	N1—Cd1—O1—S1	−41.99 (12)
N4^i^—Cd1—N1—N2	101.12 (15)	N1^i^—Cd1—O1—S1	153.99 (12)
O1—Cd1—N1—N2	−161.04 (16)	Cd1—O1—S1—O3	176.99 (11)
O1^i^—Cd1—N1—N2	−70.88 (15)	Cd1—O1—S1—O2	−53.35 (14)
N1^i^—Cd1—N1—N2	−115.61 (15)	Cd1—O1—S1—C1	60.81 (13)
N4—Cd1—N1—C6	−171.67 (16)	C2—C1—S1—O3	21.9 (2)
N4^i^—Cd1—N1—C6	−99.86 (16)	C6—C1—S1—O3	−156.48 (16)
O1—Cd1—N1—C6	−2.02 (15)	C2—C1—S1—O2	−99.96 (19)
O1^i^—Cd1—N1—C6	88.15 (16)	C6—C1—S1—O2	81.66 (17)
N1^i^—Cd1—N1—C6	43.42 (15)	C2—C1—S1—O1	142.14 (17)
C6—N1—N2—N3	−0.4 (2)	C6—C1—S1—O1	−36.25 (18)

Symmetry codes: (i) −*x*, *y*, −*z*+1/2.

### Hydrogen-bond geometry (Å, °)

**Table:**

*D*—H···*A*	*D*—H	H···*A*	*D*···*A*	*D*—H···*A*
N3—H3N···O2^ii^	0.93	1.86	2.776 (3)	167.
C3—H3···O3^iii^	0.93	2.55	3.255 (3)	133.
C7—H7···O2^iv^	0.93	2.56	3.204 (3)	127.

Symmetry codes: (ii) *x*+1, *y*, *z*; (iii) −*x*−1/2, −*y*−1/2, −*z*; (iv) −*x*, *y*, −*z*+1/2.
